# Supplementary material for: JNK1/2 represses Lkb1-deficiency-induced lung squamous cell carcinoma progression
Source: Nat Commun. 2019 May 14;10:2148. doi: 10.1038/s41467-019-09843-1 (PMC6517592; doi:10.1038/s41467-019-09843-1)
Supplement: Supplementary file 1 — Supplementary Information [file 41467_2019_9843_MOESM1_ESM.pdf]

Supplementary Information

Liu et al. JNK1/2 represses *Lkb1*-deficiency-induced Lung Squamous Cell Carcinoma Progression

Supplementary Figures

Supplementary Fig. 1 | Characterization of lung squamous cell carcinoma (LSCC) development stages in *Lkb1*$^{d/d}$ mice; Related to Fig. 1 and Table 1.

Supplementary Fig. 2 | Characterization of lung adenocarcinoma in different mouse models; Related to Table 1.

Supplementary Fig. 3 | Characterization of *Lkb1*$^{d/d}$ mouse lung SCC transcriptome profiles and lung tumors in *Lkb1*$^{d/d}$*Pten*$^{d/d}$ mice; Related to Fig. 1 and 2 and Table 1.

Supplementary Fig. 4 | Gene set enrichment analysis (GSEA) analysis of mouse lung tumors and kinome array analysis of mouse lungs; Related to Fig. 2 and Table 1.

Supplementary Fig. 5 | MKK7 positively regulates p-JNK1/2 in human bronchial epithelial and squamous cell carcinoma cells; Related to Fig. 3.

Supplementary Fig. 6 | Analyses of cell colony formation and lung tumor development after knockout of *Jnk1/2*; Related to Fig. 4.

Supplementary Fig. 7 | Examination of the inhibition of lung tumor development after Anisomycin treatment; Related to Fig. 5.

Supplementary Fig. 8 | P-JNK1/2 expression in the human lung SCCs and the survival analyses; Related to Fig. 6.

Supplementary Fig. 9 | Full bots of the most important blots in the Figures; Related to Fig. 2, 3, 4 and 5.

Supplementary Table 1 | Top molecular and cellular functions were enriched using Ingenuity pathway analysis (IPA) of DEGs identified in the microarray analysis of 13-month-old *Lkb1*$^{d/d}$-SCC (Supplementary Data 3); Related to Fig. 1 and Table 1.

Supplementary Table 2 | Summary of *Lkb1*$^{d/d}$*Pten*$^{d/d}$ mouse lung phenotypes; Related to Fig. 2 and Table 1.

Supplementary Table 3 | Top enriched upstream regulators were identified from these differentially expressed genes identified in the microarray analysis of 3-month-old *Lkb1*$^{d/d}$*Pten*$^{d/d}$-(SCC + ASC) (Supplementary Data 3) using Ingenuity Pathway Analysis (IPA); Related to Fig. 2 and Table 1.

Supplementary Table 4 | Gene set enrichment analysis (GSEA) of enriched pathways in mouse lung tumors; Related to Fig. 1 and 2 and Table 1.

Supplementary Table 5 | Summary table of quantification results of kinome array using ImageJ; Related to Fig. 2.

Supplementary Table 6 | Calculation of the bands of p63 and ΔNp63 in Fig. 4e; Related to Fig. 4e.

Supplementary Table 7 | List of the DEGs of p63 pathway, which are identified by IPA in Table 5. Genes labelled in red were upregulated while those labelled in blue were downregulated; Related to Fig. 4.

Supplementary Table 8 | The LSCC patients' information of tissue array from Baylor College of Medicine; Related to Fig. 6 and Supplementary Fig. 8.

Supplementary Table 9 | Sequence list of SYBR primers and gRNAs, Related to Fig. 3-5 and Supplementary Fig. 1, 5 and 6.

Supplementary Fig. 1 | Characterization of lung squamous cell carcinoma (LSCC) development stages in *Lkb1*$^{d/d}$ mice; Related to Fig. 1 and Table 1.

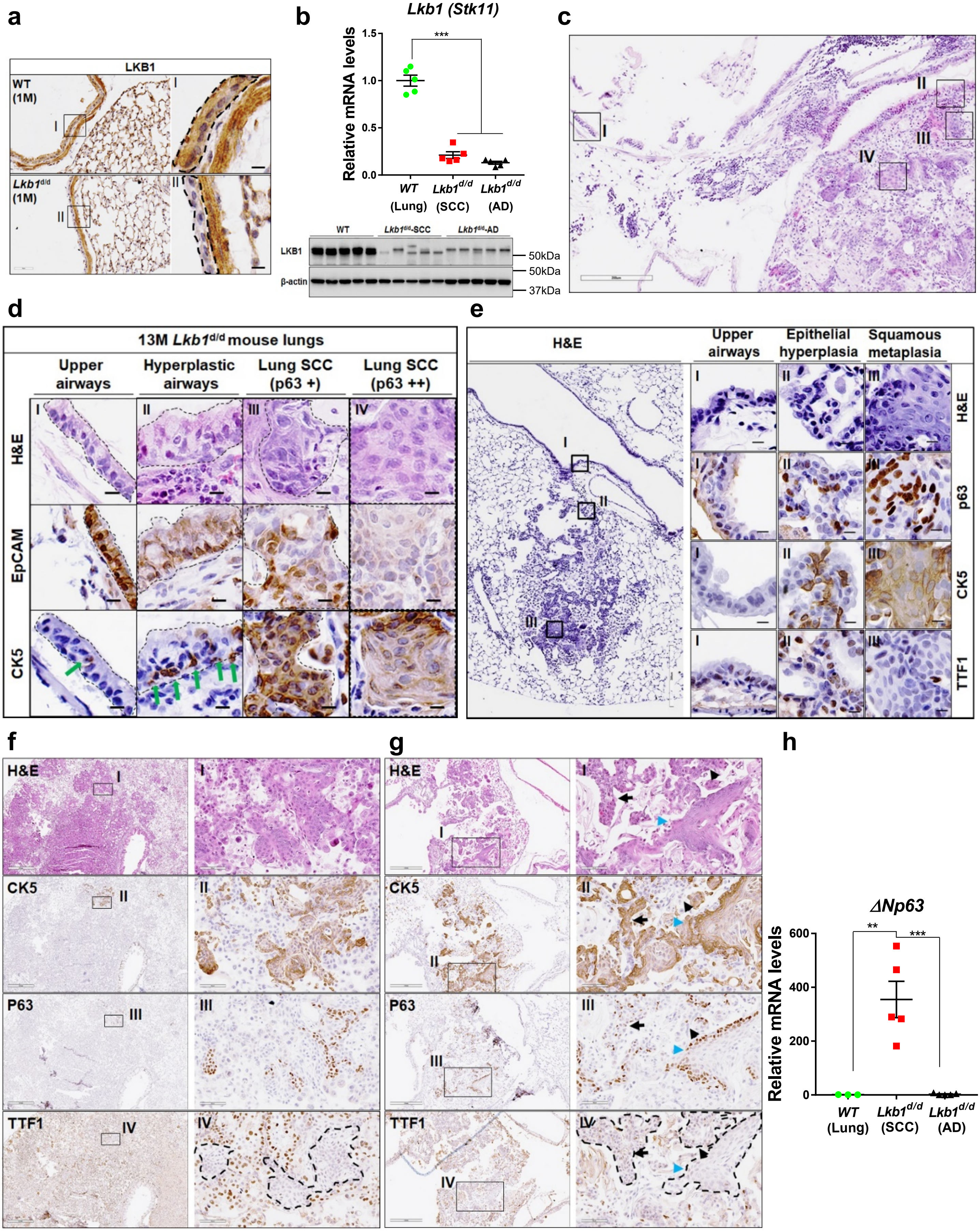

Supplementary Information

Supplementary Figures

Supplementary Fig. 1 | Characterization of lung squamous cell carcinoma (LSCC) development stages in *Lkb1*$^{d/d}$ mice; Related to Fig. 1 and Table 1.

a, Representative immunohistochemistry analysis of protein expression in 1-month-old (1M) mouse lungs (n = 6). **b,** qRT-PCR (upper panel) and Western Blot (WB) (lower panel) analysis of mRNA and protein expression of LKB1 in 13-month-old mouse lungs and lung tumors, respectively (n = 5). Error bar is SEM; T test: *** P<0.001. SCC: squamous cell carcinoma; AD: Adenocarcinoma. **c,** Representative 13-month-old *Lkb1*$^{d/d}$ mouse lung SCC (H&E). It includes the representative subareas shown in Fig. 1b. Box I: Upper airways; Box II: Hyperplastic airways; Box III: Lung SCC (p63 +); Box IV: Lung SCC (p63 ++). Scale bar: 200μm. **d,** Representative histopathology sections (H&E) and immunohistochemistry (IHC) staining of 13-month-old *Lkb1*$^{d/d}$ mouse lung SCC. These subgroups of each H&E or marker staining were from the same staining slide, respectively (e.g., four H&E subgroups are indicated in the large area of Supplementary Fig. 1c). EpCAM: A marker of epithelial cells; SCC markers: CK5. CK5 is cytoplasmic staining and diffuse. Squamous cell carcinomas showed positive staining of CK5. Within the SCCs, the cells showing intercellular bridging and keratinization tended to show positivity for CK5. "+" means the expression is mild or moderate, and "++" means the expression is strong. Dashes outline: Regions of interest. Positive staining is indicated in some panels by green arrows. Scale bar: 10μm. **e,** Representative H&E and immunohistochemistry (IHC) staining results of lung epithelial hyperplasia with squamous metaplasia in a 13-month-old *Lkb1*$^{d/d}$ mouse. The areas labelled by boxes are magnified as showing the increased expression of CK5 and p63 from airway to squamous metaplasia. Box I: Upper airways; Box II: Epithelial hyperplasia; Box III: squamous metaplasia. EpCAM: A marker of epithelial cells; SCC markers: p63 and CK5; AD marker: TTF1. Scale bar: 300μm (Black box) and 10μm (solid black). **f,** Representative H&E and IHC staining of adenocarcinoma (AD) with squamous differentiation (SqD) in a 13-month-old *Lkb1*$^{d/d}$ mouse. The

areas labelled by boxes are magnified as showing the SqD cells (I) with positive staining of CK5 in the keratinizing cells (II) and p63 in the basal cells (III), and negative staining of TTF1 (IV) which is in the areas staining positive for CK5 and P63 and is also pointed by dash outlines. Scale bar: 600μm (Black box, left panel) and 60μm (Black box, right panel). **g,** Representative H&E and IHC staining results of lung adenosquamous carcinoma (ASC) in a 13-month-old *Lkb1*$^{d/d}$ mouse. The areas labelled by boxes are magnified as showing the ASC cells. The neoplasm has both adenocarcinoma (AD) component (black arrows) and squamous cell carcinoma (SCC) component (Blue arrowhead). Many AD cells had positive staining for CK5 (II) or p63 (III) while having negative or weak staining for TTF1 (IV). SCC cells had positive staining of CK5 (II) and p63 (III) while having negative staining for TTF1 (IV). Dash outlines pointed the areas of interest. Scale bar: 600μm (Black box, left panel) and 60μm (Black box, right panel). **h,** qRT-PCR analysis of gene expression in 13-month-old mouse lungs and lung tumors (n = 5). Error bar is SEM; T test: ** P < 0.01; *** P < 0.001.

Supplementary Fig. 2 | Characterization of lung adenocarcinoma in different mouse models; Related to Table 1.

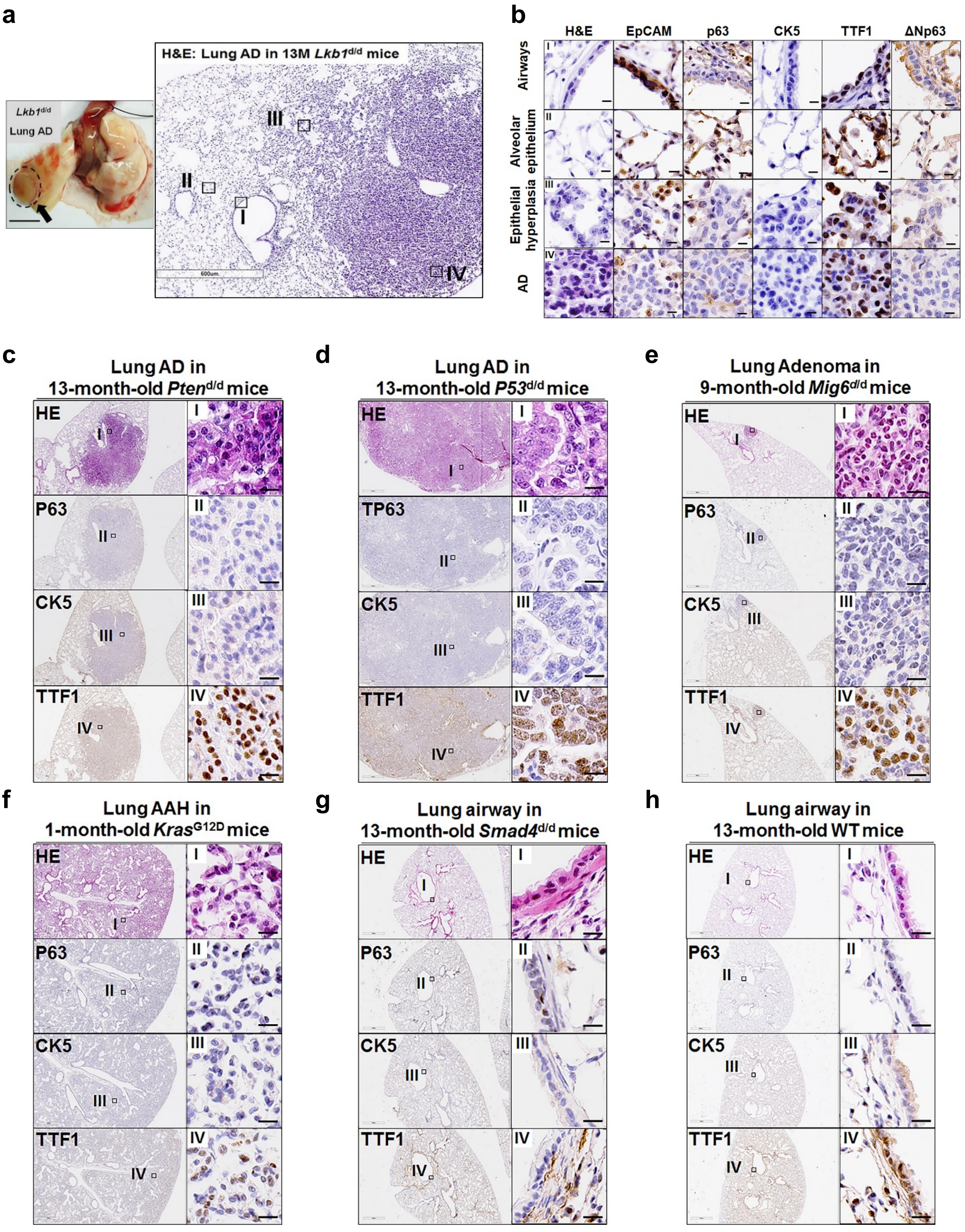

Supplementary Fig. 2 | Characterization of lung adenocarcinoma in different mouse models; Related to Table 1.

a, Gross appearance of and representative H&E and IHC staining of 13-month-old *Lkb1*$^{d/d}$ mouse lung AD. Left panel: Gross appearance of lung tumors in 13-month-old *CCSP*^{iCre}*Lkb1*$^{f/f}$ (*Lkb1*$^{d/d}$) mice. AD was labelled by a dash cycle and an arrow. Scale bar: 0.5cm. Right panel: Representative 13-month-old *Lkb1*$^{d/d}$ mouse lung AD (H&E). Box I: Airways; Box II: Alveolar epithelium; Box III: Epithelial hyperplasia (EH); Box IV: AD; Scale bar: 600μm. **b,** These subgroups of each H&E or marker staining were from the same staining slide, respectively (e.g., four H&E subgroups are indicated in the large area of right panel); EpCAM: A marker of epithelial cells; SCC markers: p63, ΔNp63 and CK5; AD marker: TTF1; Scale bar of each area: 10μm. **c-h,** Representative H&E and IHC staining of lungs in 13-month-old *Pten*$^{d/d}$ mice (**c**), in 13-month-old *P53*$^{d/d}$ mice (**d**), in 9-month-old *Mig6*$^{d/d}$ mice (**e**), in 1-month-old *Kras*^{G12D} mice (**f**), in 13-month-old *Smad4*$^{d/d}$ mice (**g**) and in 13-month-old wild type (WT) mice (**h**). Scale bars: 600μm (Black box) and 15μm (dark bar).

Supplementary Fig. 3 | Characterization of *Lkb1*$^{d/d}$ mouse lung SCC transcriptome profiles and lung tumors in *Lkb1*$^{d/d}$*Pten*$^{d/d}$ mice; Related to Fig. 1 and 2 and Table 1.

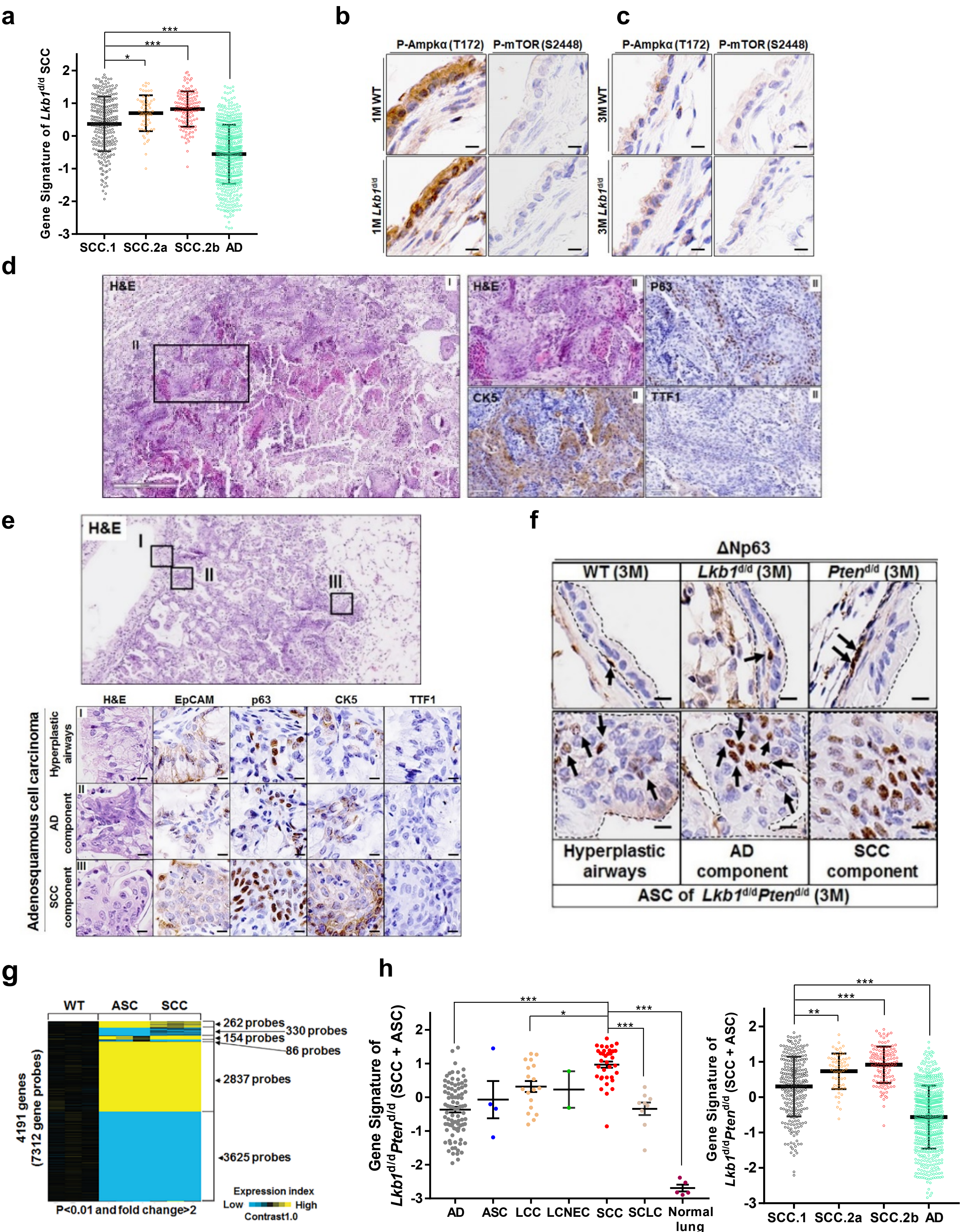

Supplementary Fig. 3 | Characterization of *Lkb1*$^{d/d}$ mouse lung SCC transcriptome profiles and lung tumors in *Lkb1*$^{d/d}$*Pten*$^{d/d}$ mice; Related to Fig. 1 and 2 and Table 1.

a, Distribution of the alignment of the gene signature of 13-month-old *Lkb1*$^{d/d}$ mouse lung tumors with TCGA human lung tumors (SCC.1, n = 259; SCC.2a, n = 62; SCC.2b, n = 123; AD.1, n = 579). The gene signatures were generated using the differentially changed genes (DEGs) identified in the microarray analysis of 13-month-old *Lkb1*$^{d/d}$-SCC (Supplementary Data 3). ANOVA (Tukey-Kramer Multiple Comparisons (TKMC) Test) was performed and error bar is SEM; * $P < 0.05$; *** $P < 0.001$. **b and c,** Representative IHC analysis of protein expression in 1-month-old (1M) (**b**) and 3-month-old (3M) (**c**) mouse lungs (n = 6). **d,** Representative H&E and IHC staining of lung SCCs in 3-month-old *Lkb1*$^{d/d}$*Pten*$^{d/d}$ mice. SCC markers: p63 and CK5; AD marker: TTF1. Scale bar: 600μm (Black box, upper panel) and 60μm (Black box, lower panel). **e,** Representative H&E and IHC staining of lung adenosquamous carcinoma (ASC) in 3-month-old *Lkb1*$^{d/d}$*Pten*$^{d/d}$ mice (n = 6). EpCAM: A marker of epithelial cell; Box I: Hyperplastic airway; Box II: Adenocarcinoma (AD) component; Box III: Peripheral squamous cell carcinoma (SCC) component; Scale bar: 100μm. These subgroups of each H&E or marker staining were from the same staining slide, respectively (e.g., four H&E subgroups are indicated in the large area of Supplementary Fig. 3e (upper panel)). SCC markers: p63 and CK5; AD marker: TTF1; Scale bar of each area: 10μm. Similar to the observation of ASCs in *Lkb1*$^{d/d}$ mice (Supplementary Fig. 1g), the portions of ASCs consisting of AD cells also exhibited the same marker expression of SCC cells in *Lkb1*$^{d/d}$*Pten*$^{d/d}$ mice (Supplementary Fig. 3g). **f,** Representative ΔNp63 IHC staining (focal and nuclear staining) of 3-month-old (3M) mouse lungs and lung tumors (n = 6). AD: Adenocarcinoma; Dashes outline: Regions of interest. Arrows were used to point to nuclear staining of P63; Scale bar: 10μm**. g,** Heatmap of the probes of differentially expressed genes (DEGs) between tumors (ASC, n= 3, or SCC, n= 3) and wild type (WT, n= 3) lungs. The detailed information is supplied in Supplementary Data 3. **h,** Gene signature analysis of the differentially changed genes identified in the microarray analysis of 3-month-old *Lkb1*$^{d/d}$*Pten*$^{d/d}$-(SCC + ASC)

(Supplementary Data 3). The left panel was analyzed as similar as that of Fig. 1c using Takeuchi human lung cancer database (GSE11969) and the right panel was performed as Supplementary Fig. 3a using TCGA human lung cancer database.

Supplementary Fig. 4 | Gene set enrichment analysis (GSEA) analysis of mouse lung tumors and kinome array analysis of mouse lungs; Related to Fig. 2 and Table 1.

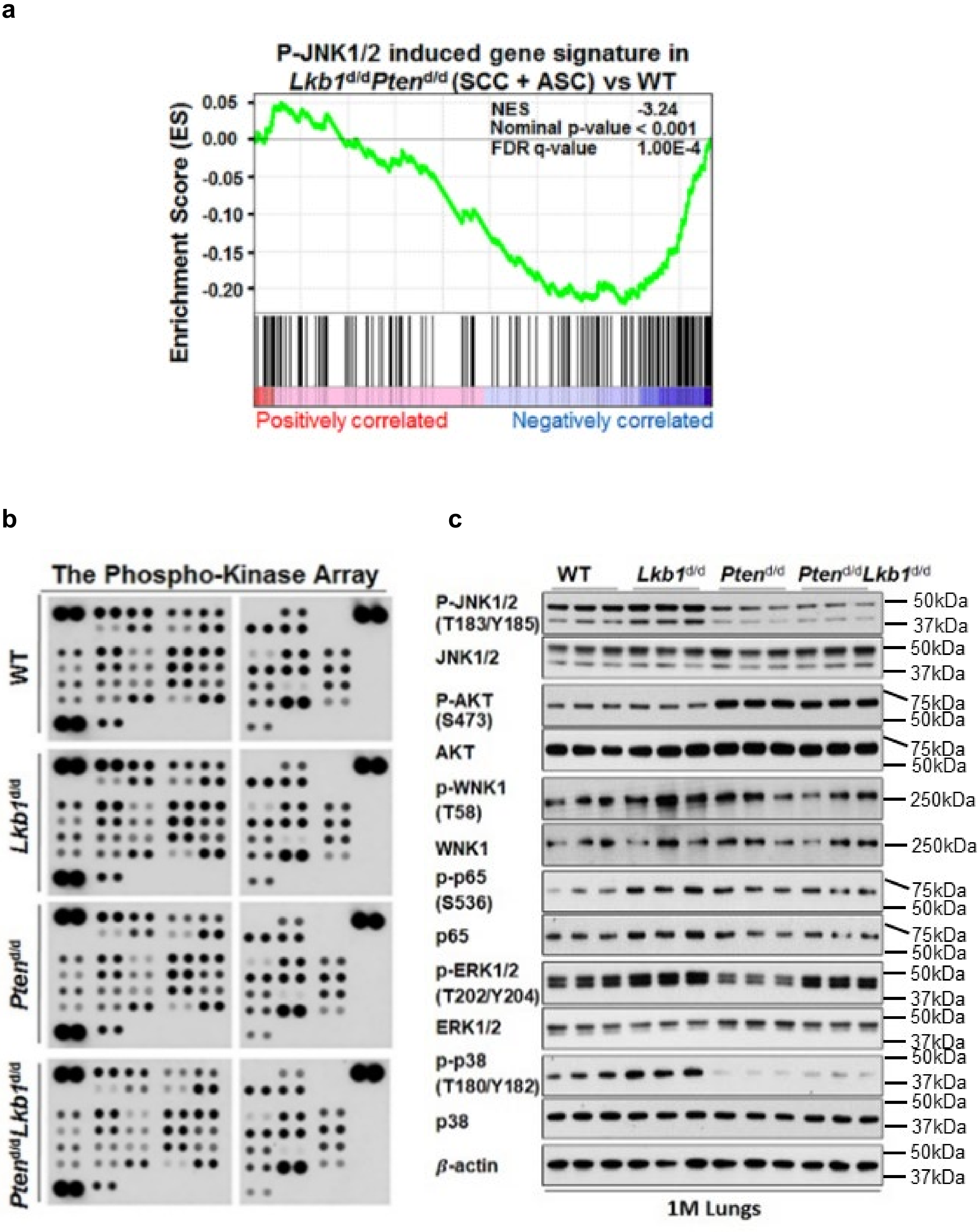

Supplementary Fig. 4 | Gene set enrichment analysis (GSEA) analysis of mouse lung tumors and kinome array analysis of mouse lungs; Related to Fig. 2 and Table 1.

a, GSEA of pre-ranked genes detected in microarrays analysis of 3-month-old *Lkb1*$^{d/d}$*Pten*$^{d/d}$ mouse lung SCC and ASC tumors. All detected genes (Supplementary Data 3) were ranked based on their fold changes (*Lkb1*$^{d/d}$*Pten*$^{d/d}$-(SCC + ASC) vs WT) from high (the left terminal of X-axis) to low (the right terminal of X-axis). ASC: Adenosquamous cell carcinoma. **b-c,** Summary of kinome array performed in 1-month-old (1M) mouse lungs. (**b**) Full map of kinome array performed in 1-month-old mouse lungs; (**c**) WB analysis of candidate pathways identified in kinome array using 1-month-old (1M).

Supplementary Fig. 5 | MKK7 positively regulates p-JNK1/2 in human bronchial epithelial and squamous cell carcinoma cells; Related to Fig. 3.

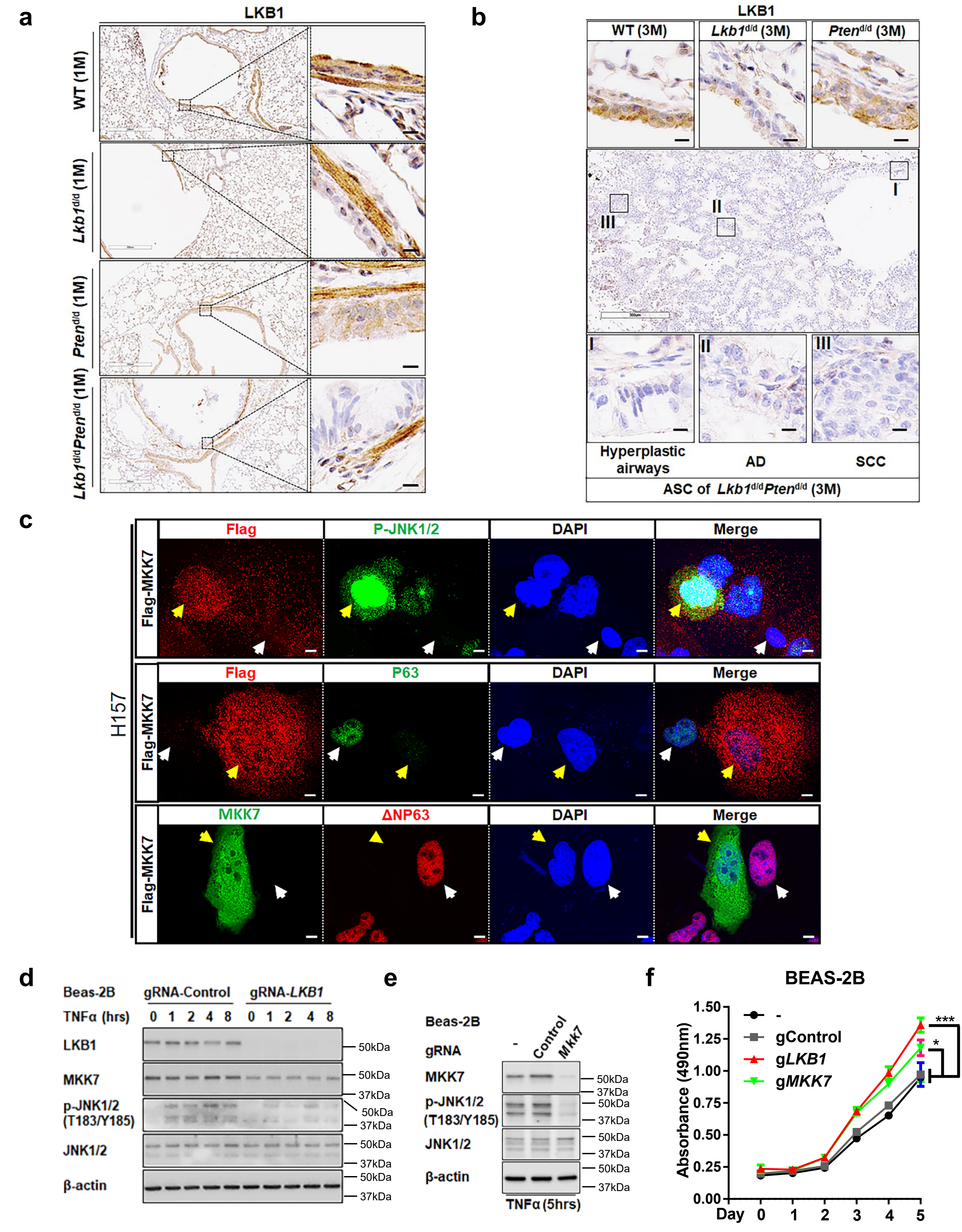

Supplementary Fig. 5 | MKK7 positively regulates p-JNK1/2 in human bronchial epithelial and squamous cell carcinoma cells; Related to Fig. 3.

a-b, Representative IHC staining of LKB1 in 1-month-old mouse lungs (n = 5) (**a**) and in 3-month-old mouse lungs and lung tumors (n = 6) (**b**). Box I: Hyperplastic airway; Box II: Adenocarcinoma (AD) component; Box III: peripheral squamous cell carcinoma (SCC) component. Scale bar: 300μm (Black box) and 10μm (dark bar). **c**, Co-staining of Flag/MKK7 and pJNK1/2 or p63 or ΔNp63 in human LSCC cells. Scale bar: 50μm; Yellow arrows point out the cells expressing the exogenous Flag-MKK7; White arrows point out the cells without clear expression of the exogenous Flag-MKK7. **d**, WB analysis of protein expressions in Beas-2B cells, an immortalized human bronchial epithelial cell line, after knockout of *LKB1* or scramble (Control) using lentiviral Cas9/gRNA with the treatment of TNFα (20 ng/ml). **e,** WB analysis of protein expressions in Beas-2B cells after knockout of *MKK7* or scramble (Control) using lentiviral Cas9/gRNA under 5-hour (hr) treatment of TNFα (20 ng/ml). **f**, MTS assay analysis of cell viability of Beas-2B. -: parental cells; gControl: gRNA targeting non-coding region; g*LKB1*: gRNA targeting LKB1; g*MKK7*: gRNA targeting MKK7.

Supplementary Fig. 6 | Analyses of cell colony formation and lung tumor development after knockout of *Jnk1/2*; Related to Fig. 4.

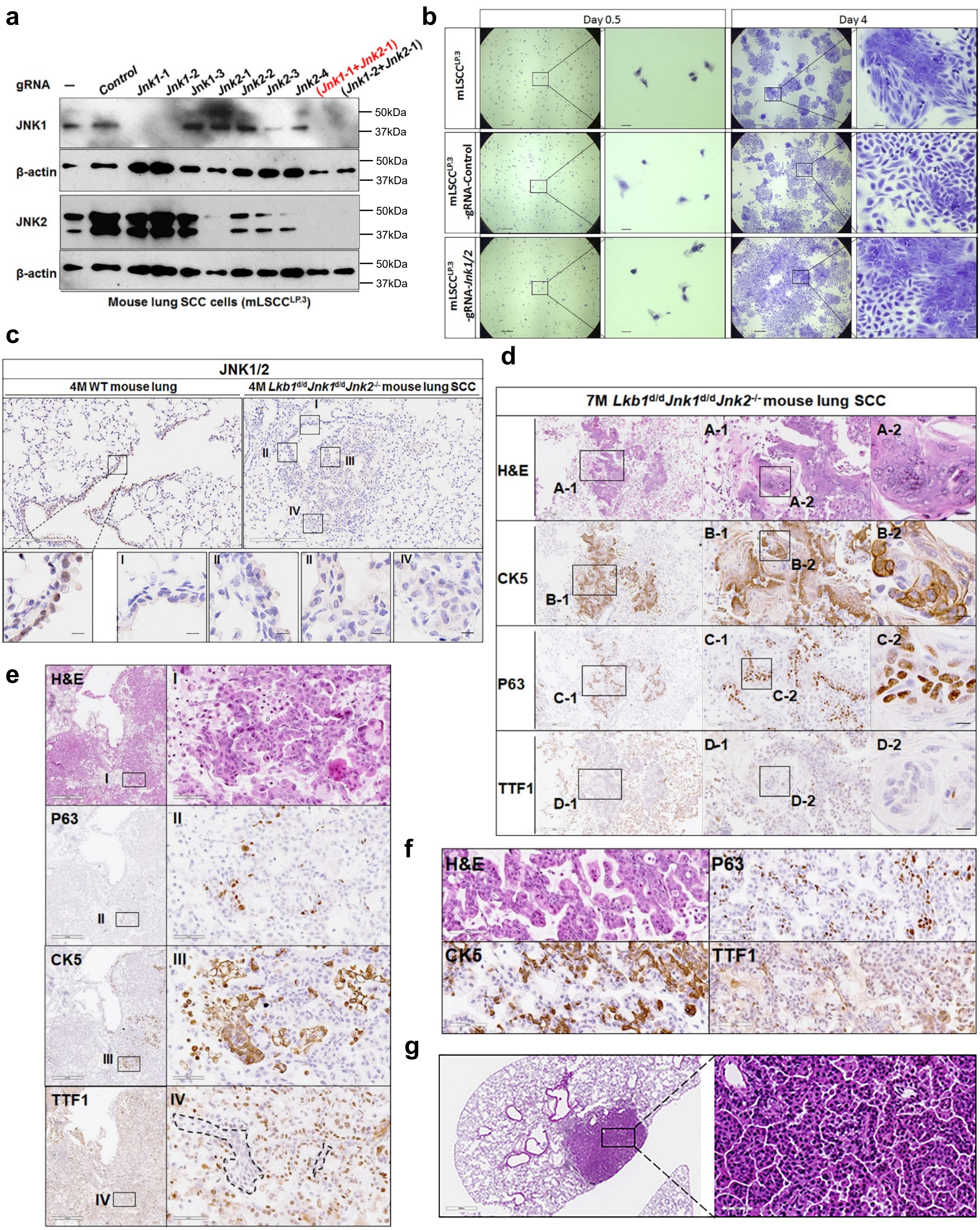

Supplementary Fig. 6 | Analyses of cell colony formation and lung tumor development after knockout of *Jnk1/2*; Related to Fig. 4.

a, WB analysis of protein levels of JNK1 and JNK2 in mLSCC$^{LP.3}$ cells, which were isolated form *Lkb1*$^{d/d}$*Pten*$^{d/d}$ mouse lung SCCs, after knockout of *Jnk1/2* or scramble (Control) using Lentiviral Cas9/gRNA. **b,** Representative crystal staining of cell colony formation of mLSCC$^{LP.3}$ cells after knockout of *Jnk1/2* or scramble (Control) and six random regions were chosen for the semi-quantification (Fig. 4a) using ImageJ. **c,** Representative IHC staining of JNK1/2 in 4-month-old (4M) WT mouse lungs and *Lkb1*$^{d/d}$*Jnk1*$^{d/d}$*Jnk2*$^{-/-}$ mouse lung pre-neoplastic lesions. Sub-areas (I-IV) were magnified. Scale bar: 200μm (Black box) and 10μm (dark bar). **d,** Representative H&E and IHC staining of 7-month-old (7M) *Lkb1*$^{d/d}$*Jnk1*$^{d/d}$*Jnk2*$^{-/-}$ mouse lung SCC. Scale bar: 300μm (Black box, left panel), 60μm (Black box, middle panel) and 10μm (dark bar). SCC markers: p63 and CK5; AD marker: TTF1. **e,** Representative H&E and IHC staining of lung adenocarcinoma with squamous differentiation in 7-month-old *Lkb1*$^{d/d}$*Jnk1*$^{d/d}$*Jnk2*$^{-/-}$ mice. The areas labelled by boxes are magnified as showing the squamous differentiation cells (I) with positive staining of CK5 in the keratinizing cells (II) and p63 in the basal cells (III), and negative staining of TTF1 (IV) which is in the areas staining positive for CK5 and is also pointed by dash outlines. Scale bar: 600μm (Black box, left panel) and 60μm (Black box, right panel). **f,** Representative H&E and IHC staining of lung adenocarcinoma (AD) expressing the typical SCC markers CK5 and p63 in 7-month-old *Lkb1*$^{d/d}$*Jnk1*$^{d/d}$*Jnk2*$^{-/-}$ mice. These areas showed the AD cells with positive staining of CK5 and p63 while also having with the weak staining of TTF1 (TTF1 is nuclear staining, and the cytoplasmic staining is background.). Scale bar: 60μm (Black box). **g**, Representative H&E staining of lung adenocarcinoma in 12-15-month-old *Jnk1*$^{d/d}$*Jnk2*$^{-/-}$ mice (Supplementary Data 1). Scale bar: 500μm (Black box, left panel); 50μm (Black box, right panel).

Supplementary Fig. 7 | Examination of the inhibition of lung tumor development after Anisomycin treatment; Related to Fig. 5.

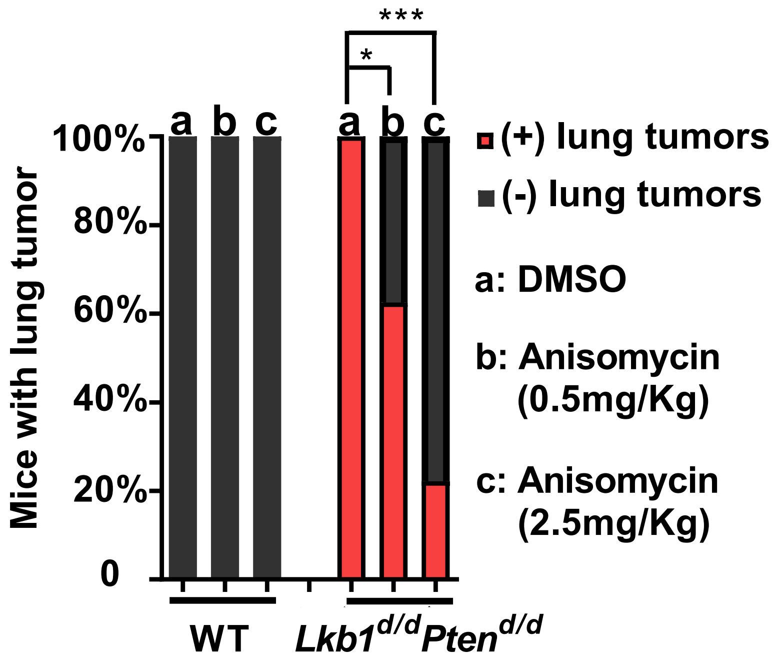

Supplementary Fig. 7 | Examination of the inhibition of lung tumor development after Anisomycin treatment; Related to Fig. 5.

Quantification of mice with lung tumors at the endpoint of treatment (Fig. 5d). The b group of *Lkb1*$^{d/d}$*Pten*$^{d/d}$ had eight mice and the remainder of each group included nine mice. $\chi 2$ test (Two-way): * $P < 0.05$; ** $P < 0.01$.

Supplementary Fig. 8 | P-JNK1/2 expression in the human lung SCCs and the survival analyses; Related to Fig. 6.

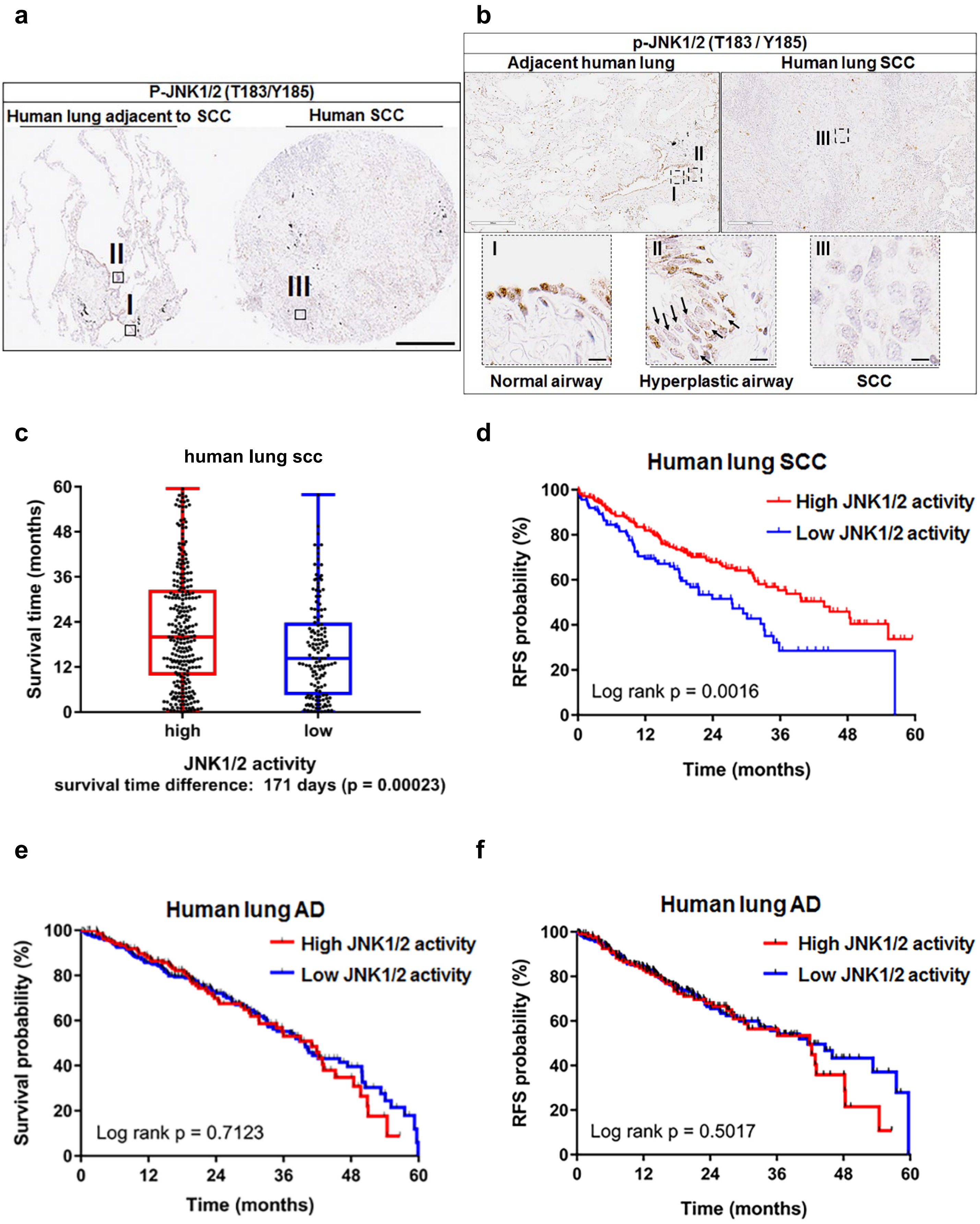

Supplementary Fig. 8 | P-JNK1/2 expression in the human lung SCCs and the survival analyses; Related to Fig. 6.

a, Representative IHC staining of P-JNK1/2 in human lung SCC and its adjacent lung. Box I: Airway; Box II: Hyperplastic airway; Box III: SCC. Scale bar: 600μm. **b,** Representative IHC staining of P-JNK1/2 in human lung SCC and its adjacent lung. Box I: Airway; Box II: Hyperplastic airway; Box III: SCC. Scale bar: 300μm (Black box) and 10μm (dark bar). Arrows: Weak nuclear staining. **c,** Boxplots of the different survival time of human lung SCC patients. *Jnk1/2* knockout mouse SCC signature score was calculated using the DEGs identified after knockout of *Jnk1/2* in $MSCC^{LP.3}$ cells (Supplementary Data 3). A patient's gene signature score was generated after *Jnk1/2* knockout mouse SCC signature was applied to TCGA human LSCC tumors. High JNK1/2 activity was defined as that the patient's gene signature score was lower than 0 and vice versa. Each small black dot represents one patient and T test was performed. **d,** Prediction of human LSCC patient relapse-free survival (RFS) rate using *Jnk1/2* knockout mouse signature. RFS tells how well a new treatment works. Each small bar of curve line represents one patient and T test was performed. **e,** *Jnk1/2* mouse signature was applied to TCGA human lung adenocarcinoma (AD) tumors. It did not predict human lung AD patient survival rate. **f,** *Jnk1/2* mouse signature was applied to TCGA human lung AD tumors. It did not predict human lung AD patient RFS.

Supplementary Fig. 9 | Full bots of the most important blots in the Figures; Related to Fig. 2, 3, 4 and 5.

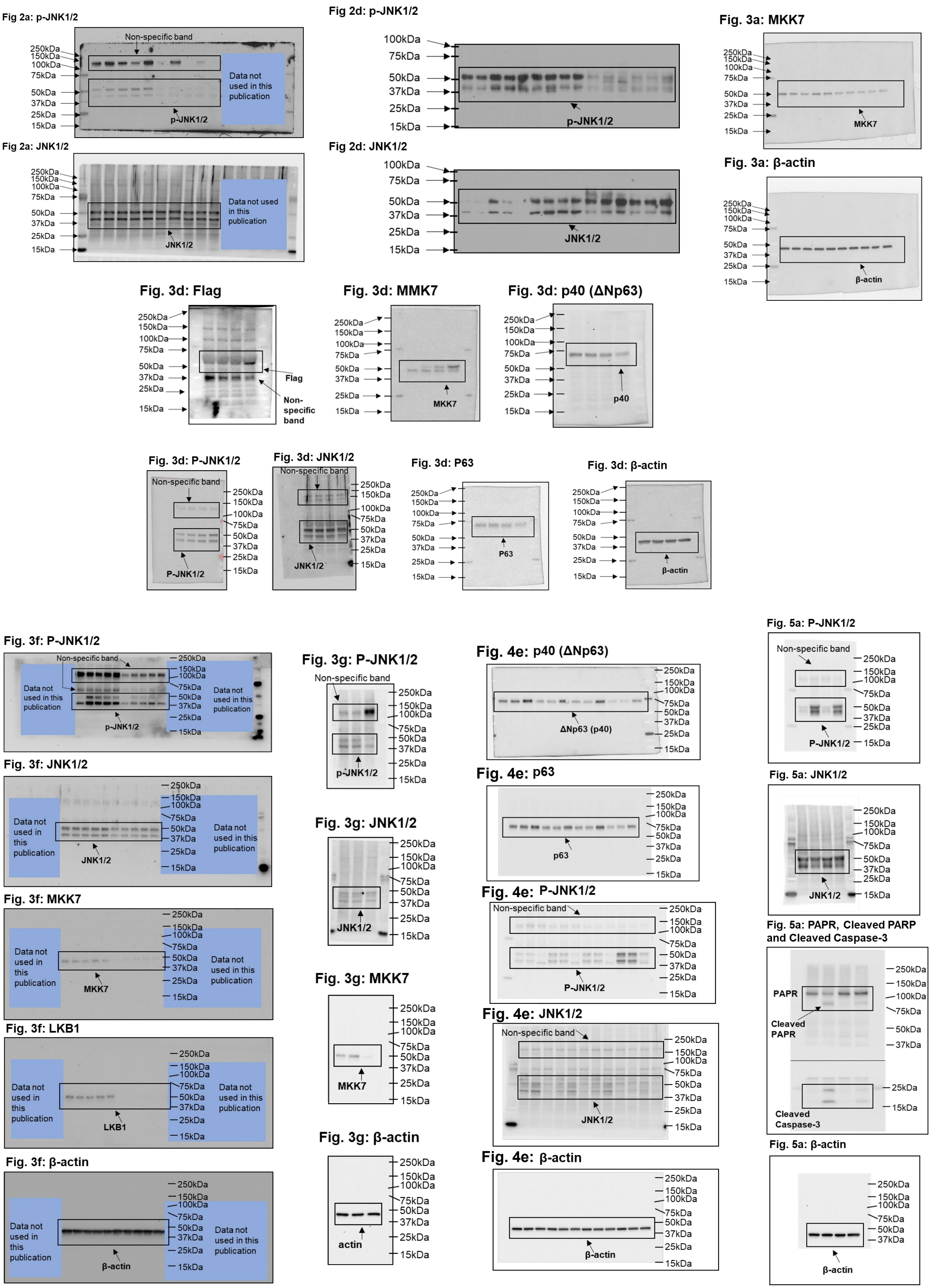

Supplementary Fig. 9 | Full bots of the most important blots in the Figures; Related to Fig. 2, 3, 4 and 5.

Supplementary Table 1 | Top molecular and cellular functions were enriched using Ingenuity pathway analysis (IPA) of DEGs identified in the microarray analysis of 13-month-old *Lkb1*$^{d/d}$-SCC (Supplementary Data 3); Related to Fig. 1 and Table 1.

**Top Molecular and Cellular Functions**	**P-value**	**#Molecules**
Cellular Movement	7.58E-09 - 6.28E-46	854
Cell Morphology	6.54E-09 - 2.27E-28	889
Cell Death and Survival	6.06E-09 - 2.03E-23	1116
Cell-To-Cell Signaling and Interaction	7.08E-09 - 1.09E-22	667
Cellular Assembly and Organization	7.16E-09 - 5.56E-18	674

Supplementary Table 2 | Summary of *Lkb1*$^{d/d}$*Pten*$^{d/d}$ mouse lung phenotypes; Related to Fig. 2 and Table 1.

**Genotype**	**No.**	**Age (Month)**	**The Percent (%) of Phenotypes in Mouse Lungs**					
			**SCC-DS**	**SCC**	**SCC-DS + SCC**	**AD-DS**	**AD**	**AD-DS + AD**
Lkb1$^{d/d}$*Pten*$^{d/d}$	9	3M	**88.9**	**88.9**	**100**	22.2	33.3	44.4

SCC: Squamous cell carcinoma; DS: Development stage; AD: Adenocarcinoma; No.: Number

SCC development stages include epithelial hyperplasia, epithelial hyperplasia with squamous metaplasia, adenocarcinoma with squamous differentiation and adenosquamous carcinoma. Adenocarcinoma development stages include epithelial hyperplasia and adenoma. No.: Number; SCC: squamous cell carcinoma; SCC-DS: SCC development stage; AD: Adenocarcinoma; AD-DS: Adenocarcinoma development stage.

Supplementary Table 3 | Top enriched upstream regulators were identified from these differentially expressed genes identified in the microarray analysis of 3-month-old *Lkb1*$^{d/d}$*Pten*$^{d/d}$-(SCC + ASC) (Supplementary Data 3) using Ingenuity Pathway Analysis (IPA); Related to Fig. 2 and Table 1.

**Top upstream regulators**	**P-value of overlap**
TGFB1	2.17E-65
beta-estradiol	9.44E-54
**TNF**	**6.79E-41**
TP53	1.17E-39
ERBB2	9.49E-34

Supplementary Table 4 | Gene set enrichment analysis (GSEA) of enriched pathways in mouse lung tumors; Related to Fig. 1 and 2 and Table 1.

The top 10 enriched oncogenic signatures in GSEA of pre-ranked genes (Supplementary Data 3) detected in microarrays between 13-month-old (13M) *Lkb1*$^{d/d}$ (upper panel) or 3-month-old *Lkb1*$^{d/d}$*Pten*$^{d/d}$ (lower panel) mouse lung tumors and the age-matched WT lungs. All detected genes are pre-ranked based on their fold changes (tumor vs WT) from high to low. Indeed, the P-JNK1/2 induced gene signature was also in the top negatively enriched pathways.

13M *Lkb1*$^{d/d}$-SCC vs WT

**Top negatively enriched oncogenic signatures**	**The names of MSigDB gene sets**	**Size**	**NES**	**NOM p-val**	**FDR q-val**
Oncogenic KRAS induced signature	KRAS.KIDNEY_UP.V1_UP	127	-4.37	0 (P<0.001)	0
mTOR induced signature	MTOR_UP.N4.V1_DN	135	-3.52	0 (P<0.001)	0
PIGF induced signature	PIGF_UP.V1_UP	170	-3.49	0 (P<0.001)	0
Late serum response inhibited signature	CSR_LATE_UP.V1_DN	140	-3.32	0 (P<0.001)	0
AKT1 inhibited signature	AKT_UP.V1_DN	174	-3.28	0 (P<0.001)	0
cAMP inhibited signature	CAMP_UP.V1_DN	183	-3.03	0 (P<0.001)	0
Oncogenic KRAS induced signature	KRAS.600_UP.V1_UP	233	-2.91	0 (P<0.001)	1.43E-04
ATF2 inhibited signature	ATF2_UP.V1_DN	165	-2.83	0 (P<0.001)	1.25E-04
**P-JNK1/2 induced signature**	**JNK_DN.V1_DN**	**165**	**-2.76**	**0 (P<0.001)**	**2.25E-04**
LEF1 induced signature	LEF1_UP.V1_UP	169	-2.74	0 (P<0.001)	2.02E-04

**Top positively enriched oncogenic signatures**	**The names of MSigDB gene sets**	**Size**	**NES**	**NOM p-val**	**FDR q-val**
AKT1 induced signature	AKT_UP.V1_UP	158	4.10	0 (P<0.001)	0
AKT1/mTOR induced signature	AKT_UP_MTOR_DN.V1_UP	168	3.35	0 (P<0.001)	0
mTOR inhibted signature	MTOR_UP.V1_UP	159	3.24	0 (P<0.001)	0
Late serum response induced signature	CSR_LATE_UP.V1_UP	152	3.08	0 (P<0.001)	0
RPS14 induced signature	RPS14_DN.V1_DN	171	3.08	0 (P<0.001)	0
Oncogenic KRAS inhibted signature	KRAS.600_UP.V1_DN	234	3.01	0 (P<0.001)	1.55E-04
EZH2 inhibted signature	PRC2_EZH2_UP.V1_UP	166	2.92	0 (P<0.001)	1.33E-04
Oncogenic KRAS inhibted signature	KRAS.50_UP.V1_DN	37	2.76	0 (P<0.001)	1.16E-04
TP53 inhibted signature	P53_DN.V2_UP	119	2.71	0 (P<0.001)	2.28E-04
IL-15 induced signature	IL15_UP.V1_UP	161	2.69	0 (P<0.001)	2.95E-04

3M *Lkb1*$^{d/d}$*Pten*$^{d/d}$-(SCC + ASC) vs WT

Top negatively enriched oncogenic signatures	The names of MSigDB gene sets	Size	NES	NOM p-val	FDR q-val
Oncogenic KRAS induced signature	KRAS.KIDNEY_UP.V1_UP	129	-3.85	0 (<0.001)	0
Oncogenic KRAS induced signature	KRAS.600_UP.V1_UP	240	-3.52	0 (<0.001)	0
LEF1 induced sigature	LEF1_UP.V1_UP	175	-3.47	0 (<0.001)	0
AKT1 inhibited sigature	AKT_UP.V1_DN	178	-3.42	0 (<0.001)	0
Oncogenic KRAS inhibited signature	KRAS.300_UP.V1_UP	123	-3.35	0 (<0.001)	0
**P-JNK1/2 induced signature**	**JNK_DN.V1_DN**	**165**	**-3.22**	**0 (<0.001)**	**0**
cAMP inhibited sigature	CAMP_UP.V1_DN	184	-3.19	0 (<0.001)	0
mTOR induced signature	MTOR_UP.N4.V1_DN	134	-3.09	0 (<0.001)	0
Late serum response inhibited signature	CSR_LATE_UP.V1_DN	142	-2.98	0 (<0.001)	0
TGFB1 induced signature	TGFB_UP.V1_UP	174	-2.85	0 (<0.001)	0

Top positively enriched oncogenic signatures	The names of MSigDB gene sets	Size	NES	NOM p-val	FDR q-val
AKT1 induced signature	AKT_UP.V1_UP	158	4.51	0 (<0.001)	0
mTOR inhibited signature	MTOR_UP.V1_UP	158	4.15	0 (<0.001)	0
AKT1/mTOR induced signature	AKT_UP_MTOR_DN.V1_UP	170	3.62	0 (<0.001)	0
RPS14 induced signature	RPS14_DN.V1_DN	172	3.50	0 (<0.001)	0
cAMP induced signature	CAMP_UP.V1_UP	176	3.25	0 (<0.001)	0
Inhibited signature of embryonic stem cells during	ESC_V6.5_UP_LATE.V1_DN	164	3.14	0 (<0.001)	0
EZH2 inbitied signature	PRC2_EZH2_UP.V1_UP	172	3.10	0 (<0.001)	0
Mutant p53 induced signature	P53_DN.V1_UP	172	3.09	0 (<0.001)	0
Late serum response induced signature	CSR_LATE_UP.V1_UP	152	2.93	0 (<0.001)	0
IL-15 induced signature	IL15_UP.V1_UP	162	2.91	0 (<0.001)	0

Supplementary Table 5 | Summary table of quantification results of kinome array using ImageJ; Related to Fig. 2.

The average density value of each protein detected from 1M mouse lungs				
**The name of protein kinase**	**WT**	***Lkb1*$^{d/d}$**	***Pten*$^{d/d}$**	***Lkb1*$^{d/d}$*Pten*$^{d/d}$**
JNK pan (T183/Y185, T221/Y223)	1	1.052859	0.662545	0.372398027
ERK1/2 (T202/Y204, T185/Y187)	1	0.882064	0.527209	0.394981609
p38 alpha (T180/Y182)	1	1.3066	0.658162	0.397569028
EGFR (Y1086)	1	1.233808	0.774525	0.417520101
MSK1/2 (S376/S360)	1	1.045242	0.790596	0.417735829
Chk2 (T68)	1	1.024578	1.014221	0.473541898
STAT5 a/b (Y694/699)	1	1.000601	0.829341	0.50158671
Lck(Y394)	1	0.871557	0.828699	0.503437418
FAK (Y397)	1	0.659071	0.716113	0.517207943
Fgr (Y412)	1	0.815016	0.797474	0.518284239
Lyn(Y397)	1	0.960328	0.774783	0.52726489
AMPK alpha1 (T174)	1	1.191487	0.794332	0.5387254
HSP27(S78/82)	1	0.818472	0.74554	0.577033205
Hck (Y411)	1	1.027017	0.680364	0.593472043
STAT5b (Y699)	1	1.051317	0.921195	0.59697185
STAT2 (Y689)	1	0.933926	0.853888	0.602910812
P53 (S392)	1	0.951457	0.960808	0.607071853
AMPKα2(T172)	1	0.83264	0.885543	0.612023752
STAT5a (Y694)	1	0.975137	0.869941	0.61752426
Yes (Y426)	1	0.994963	0.828193	0.641445683
STAT6 (Y641)	1	1.101679	0.83744	0.66517119
PDGFRβ (Y751)	1	0.86644	0.80825	0.670192368
Src(Y419)	1	1.266175	0.819086	0.673472736
TOR(S2448)	1	1.504978	0.552326	0.67546262
p53 (S46)	1	0.966849	0.889915	0.683720361
p53 (S15)	1	0.950526	1.00728	0.698688035
p27 (T198)	1	1.002939	1.042468	0.706562892
c-Jun (S63)	1	1.128751	0.913142	0.718101024
RPAS40 (T246)	1	0.951088	0.884832	0.731117745
HSP60	1	0.87968	1.353922	0.737644642
GSK-3 alpha/beta (S21/S9)	1	0.920008	0.75263	0.737888883
Fyn (Y420)	1	1.112742	0.889486	0.751573162
TOR(S2448)	1	1.194579	0.829074	0.76691129
AKT(T308)	1	1.124204	0.995622	0.77436609
p70 S6 Kinase (T421/424)	1	1.091681	1.095568	0.817831474
RSK1/2/3 (S380/386/377)	1	1.103252	0.990341	0.867286227
STAT3 (S727)	1	1.056729	1.05676	0.880199816
PLC-γ1	1	1.182568	1.176786	0.892029611
Enos (S1177)	1	1.059258	1.16785	0.912287235
STAT3 (Y705)	1	1.126529	1.115443	0.931141316
p70 S6 Kinase (T389)	1	1.117821	1.068157	0.941608446
PYK2 (Y402)	1	1.168481	1.000761	0.962570421
β-Catenin	1	1.055577	0.855765	1.056644077
AKT(S473)	1	1.045201	1.587716	1.597629465
WNK1 (T60)	1	1.072355	1.396029	1.707527565

Supplementary Table 6 | Calculation of the bands of p63 and ΔNp63 in Fig. 4e; Related to Fig. 4e.

**The ratio of bands between Anisomycin treatment and Anisomycin non-treatment**		
	gControl	g*Jnk1/2*
ΔNp63	0.27 / 1.1 = 0.245	0.73 / 1.6 = 0.456
p63	0.48 / 1.1 = 0.436	0.8 / 1.3 = 0.615

**The percent of Anisomymin-induced decrease of ΔNp63 or p63 depending on JNK1/2**	
	(g*Jnk1/2* - gControl) / g*Jnk1/2* * 100
ΔNp63	(0.456 -0.245) / 0.456 = 46.3%
p63	(0.615 - 0.436) / 0.615 = 29.1%

Supplementary Table 7 | List of the DEGs of p63 pathway, which are identified by IPA in Table 5. Genes labelled in red were upregulated while those labelled in blue were downregulated; Related to Fig. 4.

Aen	*Cdkn1a*	*F3*	*Inhba*	*Maff*	*Serpinb2*	*Ube2h*
Apaf1	*Dll1*	*Fas*	*Itga2*	*Mdm2*	*Serpine1*	*Upk1b*
Areg	*Dok1*	*Fos*	*Itga3*	*Mmp13*	*Serpinf1*	*Vegfa*
Bax	*Dusp6*	*Foxo3*	*Itga7*	*Myc*	*Slc7a11*	
Bcl2l11	*Egfr*	*Fst*	*Itgb1*	*Mynn*	*Smad3*	
Ccnd1	*Ei24*	*Hbegf*	*Jag1*	*Notch1*	*Smurf2*	
Ccng1	*Emp1*	*Hk2*	*Junb*	*Pkp2*	*Tnfaip8*	
Ccnt2	*Epha2*	*Ier3*	*Krt7*	*Ptpn12*	*Traf4*	
Cdk17	*Ercc5*	*Il1rap*	*Lyn*	*S100a8*	***Trp63***	
Abi2	*Ccne1*	*Fanca*	*Kcnk1*	*Pold2*	*Tgfb3*	
Adamts1	*Cdc25c*	*Fasn*	*Mapk8*	*Prkag2*	*Timp3*	
Ahr	*Cdk1*	*Fgfr2*	*Mcm10*	*Prkcz*	*Tipin*	
Blm	*Cdkn1b*	*Fn1*	*Mfge8*	*Prnp*	*Tnc*	
Bmp7	*Cks2*	*G6pdx*	*Mlh1*	*Racgap1*	*Ulk2*	
Brca1	*Col4a1*	*Id1*	*Mpzl2*	*Rbbp8*	*Usp48*	
Cad	*Ddb2*	*Id3*	*Myb*	*Rpa1*	*Vdr*	
Ccna2	*Dnmt1*	*Igfbp3*	*Nt5e*	*Runx2*	*Vim*	
Ccnb1	*F2r*	*Igfbp7*	*Pcna*	*Sprr3*		

Supplementary Table 8 | The LSCC patients' information of tissue array from Baylor College of Medicine; Related to Fig. 6 and Supplementary Fig. 8.

Accession #	Slide ID tumor	Slide ID normal	Type	Race	Ethnicity	Gender	Age @ surgery	Pathological Stage	Grade	Overall stage	Smoking status	Other Treatment (post surgical)	Vital Status	Years from Surgery	Recurrence
SP10-00488	b9a554b4	7c405ff1	squam	black	not hispanic	M	66	pT1b pN0 pM0	G3	Stage IB	Current smoker >25 pack-years	unknown	unknown	>5 yrs	no follow up
SP10-01122	9b5d8494	ddc3b238	squam	white	not hispanic	M	75	pT2a pN0 pM0	G3	Stage IB	Former smoker >25 pack-years	XRT	Deceased	<2 yrs	no
SP10-04944	de4ad78e	8997166b	squam	white	not hispanic	M	48	pT3 pN1 pM0	GX	Stage IIIB	Former smoker >25 pack-years	chemo prior to surgery	Alive	>5 yrs	persistent disease
SP11-00892	ebb978e1	ef58fff1	squam	white	not hispanic	M	58	pT2bpN1pM0	G3	Stage IIB	Former smoker >25 pack-years	none	Deceased	<2 yrs	no
SP11-02469	cea76a06	04117d87	squam	not reported	not hispanic	M	66	pT2a pN0 pM0	G3	Stage IB	Former smoker >25 pack-years	none	Deceased	<2 yrs	no
SP11-04661	e73e99c7	fc85796e	squam	white	not hispanic	M	79	pT2a pN0 pM0	G3	Stage IB	Current smoker >25 pack-years	none	Alive	>5yrs	no
SP11-06980	ca0ccdf7	e684f11c	squam	white	not hispanic	M	69	pT2a pN2 pM0	G3	Stage IIIA	Former smoker >25 pack-years	carbo/Taxol	Alive	>2yrs, <5yrs	no
SP12-00500	1bd8b159	109dc455	squam	white	not hispanic	M	66	pT1a pN0 pM0	G4 (small) G3 (squam)	Stage IA	Current smoker >25 pack-years	unknown	unknown	>2yrs, <5yrs	no follow up
SP12-03245	b905791e	22a63a57	squam	white	not hispanic	M	64	pT2a pN0 pM0	G4	Stage IB	Former smoker >25 pack-years	none	Alive	>2yrs, <5yrs	no
SP12-05262	483e8f23	b3f173d2	squam	white	not hispanic	M	64	pT2b pN0 pM0	G2	Stage IIA	Former smoker >25 pack-years	cisplatnum/etoposide	Alive	>2yrs, <5yrs	no
SP12-05755	973af4d0	463174 e0	squam	white	not hispanic	M	66	pT2a pN1 pM0	G3	Stage IIA	Current smoker >25 pack-years	unknown	unknown	>2yrs, <5yrs	no follow up
SP12-10328	39ad5e8d	1b6eb393	squam	white	not hispanic	M	75	pT2a pN0 pM0	G2	Stage IB	Former Smoker >25 pack-years	none	Alive	>2yrs, <5yrs	no
SP12-12095	61b78443	3e8b2cd7	squam	white	not hispanic	M	63	pT2b pN0 pM0	G3	Stage IIA	Current smoker >25 pack-years	none	Alive	>2yrs, <5yrs	no
SP12-12635	c07aa97a	2868030d	squam	white	not hispanic	M	61	pT1b pN0 pM0	G3	Stage IA	Current smoker >25 pack-years	unknown	unknown		no follow up
SP12-12805	1baa570a	5d55a35b	squam	white	not hispanic	M	56	pT2a pN1 pM0	G3	Stage IIA	Current smoker >25 pack-years	chemo therapy with outside provider	Alive	>2yrs, <5yrs	yes
SP13-03334	c6a66bad	f3e51e69	squam	black	not hispanic	M	67	pT2a pN2 pM0	G3	Stage IIIA	Current smoker >25 pack-years	unknown	unknown		no follow up
SP14-10607	9fce6a3 T2	69607b5b	squam	white	not hispanic	M	84	pT1b pN0 pM0	G3	Stage IA	Former Smoker >25 pack-years	none	Deceased	<2yrs	no
SP14-11815	d598ae3e	73a02366	squam	white	not hispanic	M	71	pT1b pN0 pM0	G2	Stage IA	Former Smoker >25 pack-years	none	Alive	>2yrs, <5yrs	no
SP14-12328	abece7bd	6e3cac44	squam	white	not hispanic	M	71	pT1b pN0 pM0	G3	Stage IIA	Former Smoker <25 pack-years	unknown	unknown		no follow up
SP11-01344	c7147fe3	2dfdac66	squam	black	not hispanic	M	71	pT1a pN0 pM0	G3	Stage IA	never	none	Alive	>2yrs, <5yrs	no
SP11-08425	0355d67b	63a8c7f9	squam	white	not hispanic	M	65	pT1b pN0 pM1	G3	Stage IA	Current smoker >25 pack-years	carbo/Taxol	Deceased	>2yrs, <5yrs	yes, <2 yrs

Sup. Table 9 | Sequence list of SYBR primers and gRNAs, Related to Fig. 3-5 and Supplementary Fig. 1, 5 and 6.

**Oligo Name (Specie)**	**Sequence 5' to 3'**
P40(F) 1(Mouse)	ATGTTGTACCTGG AAAACAATGCC
P40(R) 1(Mouse)	CAGGCATGGC ACGGATAAC
PD-L1(F) 1(Mouse)	GACCAGCTTTTGAAGGGAAATG
PD-L1(R) 1(Mouse)	CTGGTTGATTTTGCGGTATGG
**The gRNA targeting genes**	**The sequences of gRNAs**
Mouse Mapk8 CRISPR guide RNA 1	TCGCTACTACAGAGCACCAG
Mouse Mapk8 CRISPR guide RNA 2	CCAGAGGTCATTCTCGGCAT
Mouse Mapk8 CRISPR guide RNA 3	ACCAGAGGTCATTCTCGGCA
Mouse Mapk9 CRISPR guide RNA 1	AGGCTCTCTTTGCGTGCGTT
Mouse Mapk9 CRISPR guide RNA 2	TAAGAGGACGAGTTCACGGT
Mouse Mapk9 CRISPR guide RNA 3	ACTTTAAGAGGACGAGTTCA
Mouse Mapk9 CRISPR guide RNA 4	GCGTGCGTTTGGTTCTGAAA
Human LKB1 CRISPR guide RNA	CAGGTGTCGTCCGCCGCGAA
Human PTEN CRISPR guide RNA	ACCGCCAAATTTAATTGCAG
Human MAPK8 CRISPR guide RNA	TCGCTACTACAGAGCACCCG
Human MAPK9 CRISPR guide RNA	AATGGATGCTAACTTATGTC
