# Supplementary material for: JNK1/2 represses Lkb1-deficiency-induced lung squamous cell carcinoma progression
Source: Nat Commun. 2019 May 14;10:2148. doi: 10.1038/s41467-019-09843-1 (PMC6517592; doi:10.1038/s41467-019-09843-1)
Supplement: Supplementary file 3 — Description of Additional Supplementary Files [file 41467_2019_9843_MOESM3_ESM.pdf]

Description of Additional Supplementary Files

File Name: Supplementary Data 1
Description: Classification of mouse lung pathologic phenotypes based on H&E staining; Related to Fig. 1-3 and Table 1, and Supplementary Fig. 1-4 and 6.

File Name: Supplementary Data 2
Description: Classification of mouse lung pathologic phenotypes based on H&E and immunohistochemistry staining of lung tumor marker proteins; Related to Fig. 1-3 and Table 1, and Supplementary Fig. 1-4 and 6.

File Name: Supplementary Data 3
Description: Gene lists of each microarray; Related to Fig. 1, 4, 6, and Supplementary Fig. 3, 4, 7 and 8.

File Name: Supplementary Data 4
Description: Percent of lung pathologic phenotypes in *Lkb1*$^{d/d}$*Pten*$^{d/d}$ mouse tumors; Related to Fig. 2-3 and Supplementary Fig. 3-4.

File Name: Supplementary Data 5
Description: Detail information of key reagents.
