# Supplementary material for: JNK1/2 represses Lkb1-deficiency-induced lung squamous cell carcinoma progression
Source: Nat Commun. 2019 May 14;10:2148. doi: 10.1038/s41467-019-09843-1 (PMC6517592; doi:10.1038/s41467-019-09843-1)
Supplement: Supplementary file 9 — Reporting Summary [file 41467_2019_9843_MOESM9_ESM.pdf]

nature research

Corresponding author(s): Francesco J. DeMayo

Reporting Summary

Nature Research wishes to improve the reproducibility of the work that we publish. This form provides structure for consistency and transparency in reporting. For further information on Nature Research policies, see Authors & Referees and the Editorial Policy Checklist.

Statistical parameters

When statistical analyses are reported, confirm that the following items are present in the relevant location (e.g. figure legend, table legend, main text, or Methods section).

n/a	Confirmed	
☐	☒	The exact sample size (n) for each experimental group/condition, given as a discrete number and unit of measurement
☐	☒	An indication of whether measurements were taken from distinct samples or whether the same sample was measured repeatedly
☐	☒	The statistical test(s) used AND whether they are one- or two-sided<br>*Only common tests should be described solely by name; describe more complex techniques in the Methods section.*
☐	☒	A description of all covariates tested
☐	☒	A description of any assumptions or corrections, such as tests of normality and adjustment for multiple comparisons
☐	☒	A full description of the statistics including central tendency (e.g. means) or other basic estimates (e.g. regression coefficient) AND variation (e.g. standard deviation) or associated estimates of uncertainty (e.g. confidence intervals)
☒	☐	For null hypothesis testing, the test statistic (e.g. F, t, r) with confidence intervals, effect sizes, degrees of freedom and P value noted<br>*Give P values as exact values whenever suitable.*
☒	☐	For Bayesian analysis, information on the choice of priors and Markov chain Monte Carlo settings
☒	☐	For hierarchical and complex designs, identification of the appropriate level for tests and full reporting of outcomes
☒	☐	Estimates of effect sizes (e.g. Cohen's d, Pearson's r), indicating how they were calculated
☐	☒	Clearly defined error bars<br>*State explicitly what error bars represent (e.g. SD, SE, CI)*

Our web collection on statistics for biologists may be useful.

Software and code

Policy information about availability of computer code

Data collection	Aperio AT2 Scanner; Aperio ImageScope v. 12.3.0.5056; Agilent Whole Mouse Genome 4x44 multiplex format oligo arrays (Agilent Technologies, 014868); Agilent Scanner; NanoDrop ND-1000 and Agilent Bioanalyzer; Agilent Bioanalyzer;
Data analysis	ImageJ; ANOVA (TKMC Test); Agilent Feature Extraction software (v12); OmicSoft Array Studio (Version 10); Agilent Feature Extraction Software Version 11.0.1.1; Genomics Suite Gene Expression workflow of Partek software package version 6.6 (Partek Inc., St. Louis, MO, USA); quantile normalization and log2 transformation; Ingenuity Pathway Analysis (IPA, www.ingenuity.com) based on the content of 2015-03-22; oncogenic signatures (c6.all.v6.1.symbols.gmt) of the GSEA 3.0 version; Data are expressed as mean + SD or mean + SEM. The sample size (n) represents biological replicates. Student's t test was used for comparison of two group averages. When there were more than two groups, one-way ANOVA Tukey-Kramer Multiple Comparisons (TKMC) Test was performed. GraphPad Prism version 7.02 was used to generate the survival curves and calculate the p value of log-rank (Mantel-Cox) test. Spearman correlation between gene signature and protein expression and the p value were calculated using Partek Genomics Suite 6.6 software. Group size was determined based on the results of preliminary experiments and no statistical method was used to predetermine sample size in animal studies.

For manuscripts utilizing custom algorithms or software that are central to the research but not yet described in published literature, software must be made available to editors/reviewers upon request. We strongly encourage code deposition in a community repository (e.g. GitHub). See the Nature Research guidelines for submitting code & software for further information.

Data

Policy information about availability of data

All manuscripts must include a data availability statement. This statement should provide the following information, where applicable:

- Accession codes, unique identifiers, or web links for publicly available datasets
- A list of figures that have associated raw data
- A description of any restrictions on data availability

All data generated or analyzed in this study are included here (and its Supplementary Information).

Field-specific reporting

Please select the best fit for your research. If you are not sure, read the appropriate sections before making your selection.

☒ Life sciences ☐ Behavioural & social sciences

For a reference copy of the document with all sections, see nature.com/authors/policies/ReportingSummary-flat.pdf

Life sciences

Study design

All studies must disclose on these points even when the disclosure is negative.

Sample size	Group size was determined based on the results of preliminary experiments and no statistical method was used to predetermine sample size in animal studies. For each animal experiments, the number, such n = 6, has been indicated with each figure in the manuscript.
Data exclusions	No data was excluded.
Replication	All the results haven been biologically repeated three times or more.
Randomization	Yes, all the animal were randomly to be grouped in all the experiments.
Blinding	All histopathological results were blinded to pathologists, and the evaluation reports, including the classification of different groups, are provided by pathologists. All the bioinformatic and statistical analyses were unbiasedly analyzed or confirmed by bioinformaticians. The genotyping of mice was conducted by Transnetyx. All the array experiments were unbiasedly conducted and analyzed by core facilities. All qRT-PCR experiments were performed in a blinded manner.<br>The rest of group design and outcome analysis were not performed in a blinded manner. There three reasons for the rest experiments not be performed in a blinded manner. 1> All these experiments have been recorded daily in the lab notebook and all the raw data have been securely stored in the lab or in the core labs. 2> The rest of mouse experiments wound not be compromised since mouse histopathological results were blindly evaluated by pathologists. 3> It is not feasible to perform in all routine experiments in a blinded manner.

Materials & experimental systems

Policy information about availability of materials

n/a	Involved in the study
☐	☒ Unique materials
☐	☒ Antibodies
☐	☒ Eukaryotic cell lines
☐	☒ Research animals
☐	☒ Human research participants

Unique materials

Obtaining unique materials	CCSPiCre mouse, mLSCCLP.3 cells

Antibodies

Antibodies used	EpCAM (Proteintech, 21050-1-AP), p63 (Cell Signaling, 13109), CK5 (Abcam, ab52635), TTF1 (DAKO, M3575), CK7 (Santa Cruz, sc-23876), P-JNK1/2 (Abcam, ab4821), JNK1/2 (Santa Cruz, sc-572), ΔNp63 (p40) (BioCare, 3066), LKB1 (Cell Signaling, 13031); p-JNK1/2 (Cell Signaling, 4668), JNK (Santa Cruz, sc-572), P-AKT (Cell Signaling, 4060), AKT (Cell Signaling, 4691), P-P65 (Cell Signaling, 3033), P65 (Cell Signaling, 8242), P-ERK1/2 (Cell Signaling, 4370), ERK1/2 (Cell Signaling, 4695), P-p38 (Cell Signaling, 4511), p38 (Cell Signaling, 8690), PARP (Cell Signaling, 9542), Cleaved Caspase 3 (Cell Signaling, 9664), LKB1 (Cell Signaling, 3047), P-WNK1 (Abcam, ab53137), WNK1 (Abcam, ab53151), JNK1 (Cell Signaling, 3708), JNK2 (Cell Signaling, 9258), and β-Actin (Sigma, A5441). MKK7 (Abcam, ab52618; Cell Signaling, 4172), Flag (Sigma, A5441), IF secondary antibodies (ThermoFisher Scientific, A10037, A21202, A21206 and A10042); IHC secondary antibodies (Vectorlabs, BA-1000 and BA-9200); WB secondary

antibodies (Sigma, A0545 and A9044).

Validation	All the antibodies can be validated in the published study.<br><br>These antibodies were also validated using IHC in this study: EpCAM (Proteintech, 21050-1-AP), p63 (Cell Signaling, 13109), CK5 (Abcam, ab52635), TTF1 (DAKO, M3575), CK7 (Santa Cruz, sc-23876), P-JNK1/2 (Abcam, ab4821), JNK1/2 (Santa Cruz, sc-572), ΔNp63 (p40) (BioCare, 3066), LKB1 (Cell Signaling, 13031), MKK7 (Cell Signaling, 4172). IHC secondary antibodies (Vectorlabs, BA-1000 and BA-9200).<br><br>These antibodies were also validated using WB in this study: p-JNK (Cell Signaling, 4668), JNK (Santa Cruz, sc-572), P-AKT (Cell Signaling, 4060), AKT (Cell Signaling, 4691), P-P65 (Cell Signaling, 3033), P65 (Cell Signaling, 8242), P-ERK1/2 (Cell Signaling, 4370), ERK1/2 (Cell Signaling, 4695), P-p38 (Cell Signaling, 4511), p38 (Cell Signaling, 8690), PARP (Cell Signaling, 9542), Cleaved Caspase 3 (Cell Signaling, 9664), LKB1 (Cell Signaling, 3047), P-WNK1 (Abcam, ab53137), WNK1 (Abcam, ab53151), JNK1 (Cell Signaling, 3708), JNK2 (Cell Signaling, 9258), Flag (Sigma, A5441) and β-Actin (Sigma, A5441). WB secondary antibodies (Sigma, A0545 and A9044).<br><br>These antibodies were also validated using IF in this study: MKK7 (Abcam, ab52618), p-JNK1/2 (Abcam, ab4821), p63 (Cell Signaling, 13109), ΔNp63 (p40) (BioCare, 3066), Flag (Sigma, A5441), IF secondary antibodies (ThermoFisher Scientific, A10037, A21202, A21206 and A10042).

Eukaryotic cell lines

Policy information about cell lines

Cell line source(s)	mLSCCLP.3
Authentication	mLSCCLP.3 cells were isolated from Lkb1d/dPtend/d mouse lung SCC using a Cancer Cell Isolation Kit (Affymetrix, CI0002) as per the protocol.
Mycoplasma contamination	It is negative for mycoplasma, which was confirmed by Tissue Culture core in Baylor College of Medicine.
Commonly misidentified lines (See ICLAC register)	The name of mLSCCLP.3 is "The 3rd pool cells of mouse lung squamous cell carcinoma with the deficiency of Lkb1 and Pten"

Research animals

Policy information about studies involving animals; ARRIVE guidelines recommended for reporting animal research

Animals/animal-derived materials	All strains were B6. 129 and described in detail in the Method section.

Human research participants

Policy information about studies involving human research participants

Population characteristics	The human lung cancer tissue array samples (Cat. NO.: LugS150Sur-01) shown in Fig 6b-c were purchased from US Biolab. And the other batches of array shown in Fig. 6d and Supplementary Fig. 8b were from the Pathology and Histology Core at Baylor College of Medicine. Histological examinations were confirmed by Dr. Patricia D. Castro and Dr. Michael M. Ittmann. The detail patient information can be found in Sup. Table 5.

Method-specific reporting

n/a	Involved in the study
☒	☐ ChIP-seq
☒	☐ Flow cytometry
☒	☐ Magnetic resonance imaging
